# Prior knowledge based mining functional modules from Yeast PPI networks with gene ontology

**DOI:** 10.1186/1471-2105-11-S11-S3

**Published:** 2010-12-14

**Authors:** Liping Jing, Michael K Ng

**Affiliations:** 1 School of Computer and Information Technology, Beijing Jiaotong University, Beijing, 100044, P.R. China; 2 Centre for Mathematical Imaging and Vision, and Department of Mathematics, Hong Kong Baptist University, Kowloon Tong, Hong Kong, China

## Abstract

**Background:**

In the literature, there are fruitful algorithmic approaches for identification functional modules in protein-protein interactions (PPI) networks. Because of accumulation of large-scale interaction data on multiple organisms and non-recording interaction data in the existing PPI database, it is still emergent to design novel computational techniques that can be able to correctly and scalably analyze interaction data sets. Indeed there are a number of large scale biological data sets providing indirect evidence for protein-protein interaction relationships.

**Results:**

The main aim of this paper is to present a prior knowledge based mining strategy to identify functional modules from PPI networks with the aid of Gene Ontology. Higher similarity value in Gene Ontology means that two gene products are more functionally related to each other, so it is better to group such gene products into one functional module. We study (i) to encode the functional pairs into the existing PPI networks; and (ii) to use these functional pairs as pairwise constraints to supervise the existing functional module identification algorithms. Topology-based modularity metric and complex annotation in MIPs will be used to evaluate the identified functional modules by these two approaches.

**Conclusions:**

The experimental results on Yeast PPI networks and GO have shown that the prior knowledge based learning methods perform better than the existing algorithms.

## Background

Protein-protein interactions give a fundament knowledge of the biological process within a cell. Such interactions are helpful for deciphering the molecular mechanisms underlying given biological functions. Usually, the connections between proteins can be represented on a graph in which the nodes corresponding to proteins and the edges corresponding to the interactions. There are many ways to identify protein-protein interactions, for instance, according to the proteins similarity calculated based on gene expression profile, biomedical literature, and etc, see [1,2]. In order to further investigate the topological properties and functional organizations of protein networks in cells, the discovery of complex formation (also called as functional module) from PPI networks becomes a major research topic in systems biology [3,4].

### Related work

Most previous methods [5-16] for automatic complex identification or related functional module detection have employed the unsupervised graph clustering techniques and try to discover similarly or densely connected subgraphs of nodes, e.g., Newman-Girvan method (NG) [8]. Mason and Verwoerd [17] provided an overview of recent and traditional approaches to the problem of identifying community structure in biological networks. Brohee and Heldan [18] made a comparative assessment for protein-protein interaction networks of four clustering algorithms: Markov clustering (MCL), restricted neighborhood search clustering (RNSC), super paramagnetic clustering (SPC), and molecular complex detection (MCODE). They found that MCL and RNSC were more robust to identify community structure in graph alterations than the other two algorithms.

Qi et al. [15] summarized the existing complex identification methods and divided them into five categories: graph segmentation, overlapping clustering, new similarity measures, conservation across species and spatial constraints analysis. In [7] and [8], the authors attempted to segment the PPI graph into disjoint highly connected clusters (complexes) based on the nodes’ neighboring interactions cost or the iterative edge-removal process. Since some proteins are part of multiple complexes or functional modules, a number of approaches [5,6,9,11] allow overlapping clusters. Scholtens et al. [10] applied a local modeling method to better estimate the protein complex membership from direct mass spectrometry complex data and Y2H binary interaction data. They claimed to achieve a finer level of detail than that obtained by using only the mass spectrometry data. In contrast to the divisive approach, the techniques proposed in [12] in an agglomerative fashion. Asur et al. [12] proposed an ensemble approach based on different hierarchial clustering algorithms for various vertices topological similarity metrics. They experimentally demonstrated the effectiveness of such ensemble clustering approach. There are some approaches based on analysis of the spectrum of the Laplacian or similarity matrix of the network described in [19]. Also, several works have established the interconnection between expression profile similarity and protein interactions [20,21]. Even though there are fruitful algorithmic approaches developed for dissection of interaction network, identifying functional modules correctly becomes a bottleneck in the current research. One reason is the accumulation of such large-scale interaction data on multiple organisms [1,22]. The other reason is that a large portion of protein-protein interactions are not recorded in the existing PPI database. Thus it is emergent to design novel computational techniques that will be able to correctly and scalably analyze interaction data sets. Meanwhile, besides PPI databases, there are a number of large scale biological data sets providing indirect evidence for protein-protein interaction relationships. For instance, the well-established microarray technologies provide a wealth of information on gene expression in various tissues and under diverse experimental conditions. Recently, researchers began to combine these existing biological resources to detect the previously unknown regulated modules in interaction networks. In [13,14,23,24], researchers integrated gene expression profiles and PPI networks to evaluate the weights of edges or noded in the graph. Supervised predicting functional modules based on the complex prior information and eight data sources [15].

Sohler et al. proposed a joint analysis concept for mining biological networks and expression data in [23]. By integrating much more sources (including expression profile, sequence information, PPI database and etc.), Dittrich et al. [14], Zheng et al. [24], and Ulitsky and Shamir [13] used aggregation statistic methods to re-weight the importance of each node and edge in the protein-protein interactions graph. Dittrich et al. searched the subnets in the graph with large scores as the functional modules. Zheng et al. used the general graph clustering algorithm (MCL) to mine the subgraphs. Ulitsky and Shamir proposed a statistical method to find the subnetworks by using the maximum likelihood approach.

### Prior knowledge based learning

Most existing functional modules mining methods are unsupervised and they are based on the assumption that complexes for a clique in the interaction graph. However, many complexes with other topological structures, e.g., ’star’ or ’spoke’ model exit in real applications. Yeger-Lotem et al. [25] have raised this issue in the complex identification. Qi. et al. [15] firstly adopted supervised learning for protein complex identification, but their method needs abundant prior knowledge about complexes to build a probabilistic Bayesian network as a learning model. In real applications, there may be only a few prior sufficient knowledge to build a learning model. In this case, semi-supervised learning [26] is a good way to handle the learning problem with only a few prior knowledge but with a large of unlabeled information. Usually, the prior knowledge is represented in the form of pair-wise constraints, must-link and cannot-link constraints. A must-link constraint specifies that objects pair connected by the constraint belonging to the same group, while a cannot-link constraint specifies that objects pair connected by the constraint, cannot belong to the same cluster. The semi-supervised learning method has been applied to many applications, such as text classification [27] and computer-aided diagnosis [28].

Hartwell et al. [29] defined a functional module as a discrete entity whose function is separable from those of other modules. In other words, the proteins in the same module should have similar functions. Usually, PPI networks are good resources to find protein functional modules. In PPI networks, functional modules can be taken as special kinds of subgraphs, where each subgraph is consistent of a subset of nodes with a specific set of edges connecting among proteins. Based on these knowledge, the main aim of this paper is to present a prior knowledge based learning strategy to identify functional modules from PPI networks with the aid of Gene Ontology [30]. The Gene Ontology (GO) database holds functional gene annotation in a hierarchical structure that reflects the relationship between the biological terms and associated gene products. Thus, the functional relationship between two annotated gene products can be calculated as a similarity value [31,32] according to the GO hierarchical structure. Higher similarity value means that two gene products are more functionally related to each other, so it is better to group such gene products into one functional module. Our proposed semi-supervised learning strategy can use such gene product pairs as the prior information in two ways. One is to encode these functional pairs into the data representation, i.e., combining them with the existing PPI networks. The other approach is to use these functional pairs as pairwise constraints to supervise the existing functional module identification algorithms such as MCL and MCODE. Topology-based modularity metric [8] and complex annotation in MIPs [33] will be used to evaluate the identified functional modules by the proposed approaches. The experimental results on Yeast PPI networks and GO have shown that the prior knowledge based learning methods perform better than the existing algorithms.

The rest of paper is organized as follows. In Section 2, we describe the methods calculating protein similarity based on GO, and analyze the relationship between functional similarity values and existing PPI networks. Then we propose two prior knowledge based learning methods for identifying functional modules from PPI networks with the aid of GO. Experimental results on Yeast PPI networks and Gene Ontology were described and discussed in Section 3. In Section 4, we make a conclusion and showed our future work in brief.

## Methods

### Gene products functional similarity

Quantitative measure of functional similarity between gene products has been used in many applications, eg., to validate high-throughput protein interaction, help the development of new pathway modeling tools and clustering methods and enable the identification of functionally related gene products independent of homology [32,34]. GO [30] provides a good vocabulary system to estimate the functional relationship between gene products.

In GO structure, terms and their relationships are represented in the form of directed acyclic graphs. GO-based semantic similarity measures can be classified into two categories. The first category defines semantic similarity based on GO structure. The similarity between two gene products is estimated by the number of nodes two gene products share divided by the total number of nodes in two graphs. The other category is based on information content that is defined as the frequency of each GO term occurring in an annotated data set [32]. This kind of methods assume that the more information two terms share indicated by the information content of terms, the more similar they are. In this paper, we adopted the second category to calculate the semantic similarity between gene products because it was proved to be more efficient than the former one [32].

#### Similarity measure

The relevance similarity *Sim_Rel_*[32] is calculated based on the probability of each term. The probability of a term is assumed to be its frequency *freq*(*c*) = ∑ {*occur*(*c_i_*)*|c* ∈ *Ancestor*s(*c_i_*)}. in the annotations of a databases [35] Note that, for each ancestor *a* of a concept term c, we have *freq*(*a*) *≥ freq*(*c*), because the set of descendants of *a* contains all the descendants of c. Then the probability of a term *c* is defined as *p*(*c*) = *freq*(*c*)*|freq*(*root*) where *freq*(*root*) is the frequency of the root term. The probability is calculated independently for each ontology. It is monotonically increasing as one moves up on a path from a leaf to the root.

Based on the probability *p*(*c*) of each term, the information content (i.e., the amount of information shared by terms) can be measured. Schlicker et al. [32] called this kind of information content as the Relevance similarity *Sim_Rel_* between a pair of terms. *Sim_Rel_* defines the similarity between two terms in two parts:(1)

*S*(*c*1, *c*2) is the set of common ancestors of terms *c*_1_ and *c*_2_. The first part evaluates the ratio of the commonality of the terms and the information needed to fully describe the two terms. The second part records the position information of the two terms in the whole ontology. Schlicker et al. [32] have studied many methods to compute the semantic similarity between GO terms. It has been shown in [32] that the measure in (1) can consider as much information about the terms in GO as possible. Given a gene products list *G* = {*g*_1_, *g*_2_, …, *g_n_*}, the corresponding annotation terms for each gene can be identified in GO as *AT_i_* = {*c_i_*_1_, *c_i_*_2_,…, *c_i_*_|_*_g_i__*_|_,} where |*g_i_*| is the total number of annotation terms in GO for gene product *g_i_*. Finally, the semantic similarity between two gene products *g_i_* and g*_j_* can be calculated with(2)

We used Bioconductor package SemSim in R project (http://bioconductor.org/packages/2.2/bioc/html/SemSim.html) to calculate the Yeast gene products functional similarity.

#### Comparison of protein pairs and PPIs in terms of functional similarity

**Figure 1:**
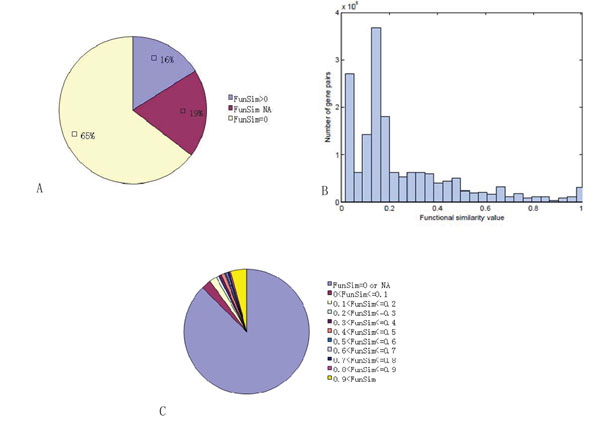
**The functional similarity value distribution of all Yeast protein pairs based on GO.** Subfigure (a) all values distribution with 10508820 pairs, (b) the > 0 values distribution with 1686196 pairs, (c)similarity distribution of MIPs Yeast PPIs.

In order to investigate the functional similarity between each pair of gene products, we calculated *Sim_Rel_*between Yeast gene products which were downloaded from SGD [36]. Meanwhile, we check the distribution of functional similarity value in recorded Yeast PPIs downloaded from MIPs database [33]). There are total 6201 Yeast proteins are included in SGD, among them, 4554 Yeast proteins are covered in MIPs database with 12316 protein protein interactions, here we ignored the self loop and direction. Based on *Sim_Rel_* (Eq.(1)) method, the functional similarity value of each gene product pair ranges from 0 to 1. A functional similarity value close to one indicates high functional similarity whereas a value close to zero indicates low similarity. We analyzed the distribution of the functional similarity value in terms of all Yeast protein pairs and MIPs Yeast PPI networks. Because there are some genes (1616) are not annotated in GO, total 4585 (6201-1616) genes as the input of SemSim package, 4585 × 4584/2 = 10508820 similarity values for the corresponding gene pairs. 65 percent of similarity values has zero value while 19 percent cannot be identified because some genes are annotated by different GO (eg., GOBP, GOCC or GOMF), the remaining 16 percent of gene product pairs has similarity value great than zero, as shown in Figure 1. Again, most of gene product pairs have smaller similarity value, which means that they are not very similar in terms of function. For the gene product pairs with similarity values close to one, we will analyze them in detail and used them as our prior information for semi-supervised mining functional modules from PPI networks. Because some genes are not annotated by GO terms, their corresponding similarity values are zero or can not be identified, and only about 10 percent of PPIs have functional similarity value greater than zero, higher similarity value means that the corresponding proteins are more similar to each other with regarding to function.

**Figure 2:**
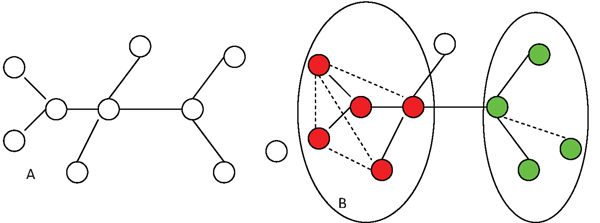
**The effect of the functional similarity on module identification.** Subfigure (a) shows the protein protein interactions, new protein relations (marked as dash line) were added to (a) because they have higher functional similarity, then a new protein network was built as show in (b). Both modules (circled) will be easily found in (b). Note that the five genes in the left cycle form a module only after the addition of the functional relations between genes, so does the right module.

The goal of functional modules identification from PPI networks is to determine a group of cellular components and their interactions attributed as specific biological functions [29]. In other words, the proteins in one module will be related to each other with regarding function. Usually, PPI networks used here are recorded in the existing PPI networks database, e.g, MIPs, where the major part of PPI information are extracted by manual annotation from the yeast literature. However, limited number of literatures make such PPI information insufficient. Therefore, identifying modules based on such insufficient PPI information (i.e., the existing PPI networks database) will not get a good performance. As shown in Figure 2a, ten proteins were listed with eight interactions which are recorded in the existing PPI networks database. In this case, it is difficult for any method to identify functional modules. Actually, the protein pairs without recorded interaction information may share common functions shown in Figure 2b marked as dash line. Once such functional information is added, it will be easy to derive the functional components for these ten proteins, finally two modules (left module with clique shape and right module with star shape) in this example are found and circled in Figure 2b.

From the example in Figure 2, we can see that the additional protein functional information is helpful for modules identification. In this paper, GO was used to obtain protein functional information indicated by the similarity value. In the next section, we will describe how to use such functional information to supervise mining functional modules from PPI networks.

### Functional modules identification methods

Before introducing our proposed functional modules identification methods, we briefly review the existing popular function modules mining algorithms, including hierarchical clustering (HC) [37], Newwan-Girvan (NG) [8], MCL [38] and MCODE [6].

#### Hierarchical clustering

In the view of computation, functional modules are special kind of subgraphs in PPI networks, and each subgraph is consistent of a subset of nodes with a specific set of edges connecting them. Meanwhile, hierarchical clustering method [37] is popularly used in networks clustering, thus, we use it to obtain the base clusters, i.e, functional modules. The implementations of this hierarchical clustering algorithm (agglomerative average-linkage hierarchical algorithm (Agnes)) is available in R project, a cluster package http://cran.r-project.org/web/packages/cluster/index.html). Agnes finds the clusters by initially assigning each object to its own cluster and then repeatedly merging pairs of clusters until either the desired number of clusters has been obtained or all of the objects have been merged into a single cluster leading to a complete agglomerative tree. The algorithm takes input as a similarity matrix. Next, we will employ two different similarity metrics, Clustering Coefficient (*S_cc_*) [3] and Neighborhood (*S_nb_*) [39] designed to capture various topological properties of scale-free networks because PPI networks are typical scale-free networks [40], and the corresponding clustering methods are called as *HC_cc_* and *HC_nb_* respectively. The first similarity metric is based on the Clustering coefficient, a popular metric from graph theory. The clustering coefficient [41] is a measure that represents the inter-connectivity of a vertex’s neighbors. The clustering coefficient of a vertex v with degree *k_v_* can be defined as follows:(3)

where *n_v_* denotes the number of triangles that go through node v. Essentially, if the edge between two nodes contributes a lot to the clustering coefficients of the nodes, then they are considered similar and should be clustered together. Here the edge-clustering coefficient [3] is defined, in analogy with the usual node-clustering coefficient, as the number of triangles to which a given edge belongs, divided by the number of triangles that might potentially include it, given the degrees of the adjacent nodes. More formally, for the edge-connecting node *i* and node *j*, the edge-clustering coefficient is(4)

where *z_i_*_,_*_j_* is the number of triangles built on that edge, i.e., the number of common neighbors of node *i* and node *j. min*[(*k_i_* – 1), (*k_j_* – 1)] is the maximal possible number of triangles.

The idea behind the use of this metric is that edges connecting nodes in different communities are included in few or no triangles and tend to have small values of *S_cc_*(*i*, *j*)*.* On the other hand, many triangles exit within clusters. Hence the coefficient *S_cc_*(i, j) is a measure of how intercommunication a link is. Note that *S_cc_*(*i*,*j*) will be zero when *k_i_* ≤ 1 or *k_j_* ≤ 1, also, when *z_i.j_* = 0.

The second metric we use is a Neighborhood-based similarity metric. We use the well-known Czekanowski-Dice distance metric [39] for this purpose. This metric uses the adjacency list of each node and favors nodes that have several common neighbors. Two nodes having no common neighbor will have the minimum similarity value (i.e. zero), while those interacting with exactly the same set of nodes will have the maximum value. The Neighborhood Based similarity metric is defined as:(5)

Here, *Int*(*i*) and *Int*(*j*) denote the adjacency list (including themselves) of proteins i and j, respectively, and Δ represents the symmetric difference between the sets. The value of this metric ranges from 0 to 1. Note that using this metric, nodes that do not interact with each other may have a non-zero similarity if they have common neighbors.

#### Newman-Girvan method

Newman and Girvan [8] first introduced edge-betweenness measure for clustering networks in sociology and ecology to obtain communities. This measure favors edges between communities and disfavors ones within communities. As pointed out by Holme et al [42] edge-betweenness uses properties calculated from the whole graph, allowing information from non-local features to be used in the clustering. Newman et al. introduced three different edge-betweenness measures, Shortest-path, Random-walk and Current-flow. In this paper, we consider the Shortest-path betweenness measure, which computes for each edge in the graph the fraction of shortest paths that pass through it. It is given by:(6)

where *SP_i_*_,_*_j_* is the number of shortest paths passing through edge *e_i_*_,_*_j_* and *SP_mαx_* is the maximum number of shortest paths passing through an edge in the graph.

*EB*(*e_i_*_,_*_j_*) denotes the shortest-path edge betweenness value of the edge between nodes *i* and *j.* The edge-betweenness of an edge is the proportion of the shortest paths that edge belongs to. NG method can be taken as a divisive clustering method. It starts with one cluster of all vertices and recursively splits the most appropriate cluster at the edges with a large edge-betweenness value. The process continues until a stopping criterion (the criterion is usually the splitting steps *s*) is achieved.

#### MCL

The Markov Cluster algorithm (MCL) [38,43] simulates a flow on the graph by calculating successive powers of the associated adjacency matrix. At each iteration, an inflation step is applied to enhance the contrast between regions of strong or weak flow in the graph. The process converges towards a partition of the graph, with a set of high-flow regions (the clusters) separated by boundaries with no flow. The value of the inflation parameter (*r*) strongly influences the number of clusters, i.e., a larger number of smaller clusters will be obtained with increasing of the inflation value (*r*). The core concept behind this method is that clusters of related nodes are densely interconnected and hence there should be more long paths between pairs of nodes belonging to the same cluster than between pairs of nodes belonging to distinct clusters. Subsequently, in [5,9,44] MCL was used to identify functionally related clusters in the protein interaction network of S. cerevisiae and Human. The experimental results indicated that the identified modules did represent functional clusters within the network. In this paper, we used the MCL package (http://www.micans.org/mcl/#source) to mine the functional modules from PPI networks.

#### MCODE

Molecular complex detection (MCODE) [6] is a method to detect densely connected regions. First it assigns a weight to each vertex corresponding to its local neighborhood density, i.e., with the core-clustering coefficient instead of the clustering coefficient for each vertex. Next, starting from the top-weighted vertex (seed vertex), it recursively moves outward, including in the cluster vertices whose weight is above a given threshold (Node Score Cutoff (*t*)). During the clustering process, new members are added only if their node score deviates from the cluster’s seed node’s score by less than the set cutoff threshold. Therefore, small cutoff values create much more smaller-size clusters and vice versa. The third stage is post-processing the above clustering results by increasing the size of the complex according to a given parameter (*f*), so that there can be overlap among the modules which have already been defined. In this paper, we used the MCODE plugin in Cytospace (http://baderlab.org/Software/MCODE) to mine the functional modules from PPI networks.

### Prior knowledge based functional modules identification methods

Given the prior information (usually as pairwise constraints), semi-supervised learning approaches [26] can be implemented in two ways. One method is to restrict the solution space based on the pairwise constraints and then find the solution consistent with the constraints for other unlabeled data, such as probabilistic models [45], hierarchical clustering [46], spectral clustering [47], and etc.. The other method is employing the prior information to learn a distance metric which can be used to computer the pairwise similarity, so that the learning methods based on similarity matrix could be adopted, such as [48,49]. The key difficulty of semi-supervised learning is how to influence an learning algorithm with the prior information. An efficient and simple method to address this challenge is encoding the prior information into the data representation and then inputting the data into an existing learning algorithm [50]. The other way is using the prior information to supervised the learning process. In this section, we will give these two methods for prior knowledge based mining function modules from protein protein interaction networks.

(a) Prior information is combined into the original data set to form a new data set, and then all existing module identification algorithms can be applied on the new data set.

(b) Prior information is used by the proposed learning algorithms:

– Semi-supervised hierarchical clustering (ssHC): prior knowledge is used to construct the transitive closure [46], and then set them as the initial clusters with the other points.

– Semi-supervised NG, Semi-supervised MCL and semi-supervised MCODE (ssNG, ssMCL and ssMCODE respectively): using NG, MCL or MCODE to group PPIs into a relatively large number of sub-modules, and then establish the connections between sub-modules according to the pairwise constraints.

**Figure 3:**
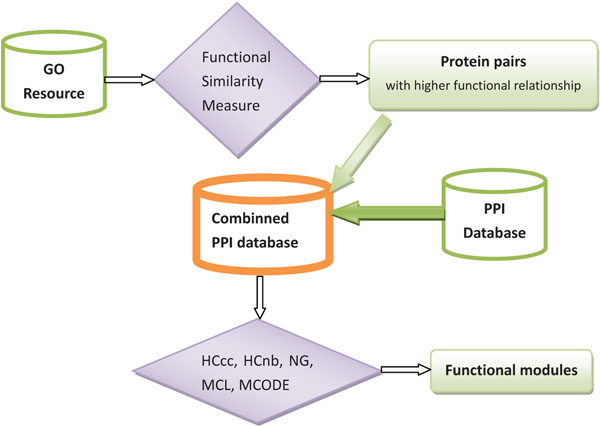
**Framework of the proposed method I**.Prior knowledge based functional modules identification methods by encoding prior information into data representation.

**Figure 4:**
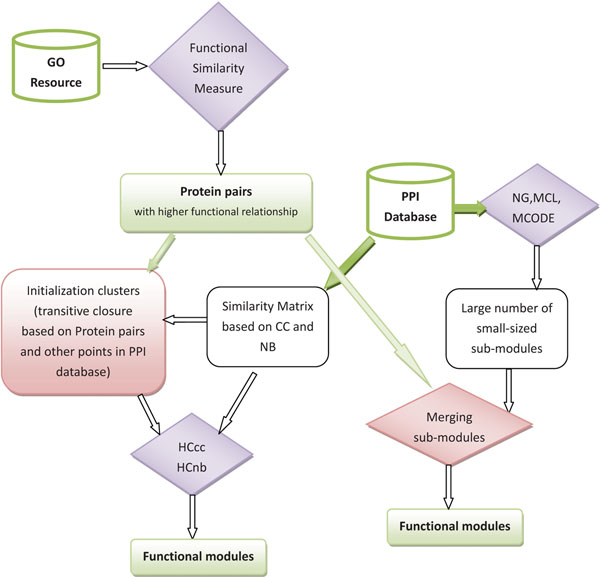
**Framework of the proposed method II.** Prior knowledge based functional modules identification methods by encoding prior information into post-processing stage of existing methods

**Figure 5:**
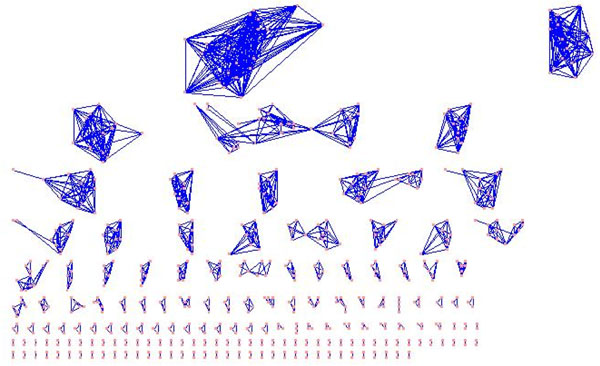
**Transitive closure examples based on protein functional pairs.** 162 transitive closures were obtained for Yeast proteins with 1488 protein functional pairs. The biggest closure has 21 proteins and 207 pairs. The smallest closure has 2 proteins and 1 pair.

The first approach (as indicated in Figure 3) encoding the prior information into the data representation is easily implemented. As shown in Figure 2, the protein functional pairs identified from GO can be added into the original PPI networks. Then, the existing functional modules identification methods (say, *HC_cc_*[3], *HC_nb_*[39], NG [8], MCL [5] and MCODE [6]) can be applied on the new combined PPI networks. Furthermore, we propose a novel prior knowledge based learning framework (as indicated in Figure 4 based on the pairwise constraints and can use any existing modules identification method, such as, *HC_cc_* , *HC_nb_*, NG, MCL, MCODE and etc.. For *HC_cc_* and *HC_nb_*, the prior information using protein functional pairs was used to construct the transitive closures [46]. The transitive closure is constructed based on the pairs of proteins with large functional similarity which gives the constraint degree between each pair of proteins. If the similarity is greater than a threshold (in this study, the best threshold is experimentally proved to be 0.999), we can say that there is a must-link constraint between *P_i_*,*P_j_*. A set of constraints *C* makes up of all the must-link constraints. In this case, an undirected graph *G*, with one node for each point appearing in the constraints *C*, and an edge between two nodes if the corresponding points appear together in a must-link constraint. Then, the connected components of *G* give the sets in the transitive closure. For instance, in our example in last Section, there are 162 transitive closures on 654 Yeast proteins with 1488 protein functional pairs, where different closures may cover different numbers of proteins. The biggest closure has 21 proteins and 207 pairs, while the smallest closure has 2 proteins and 1 pair, as shown in Figure 5.

Such transitive closures and the other proteins which are not included in these closures will be set as the initial clusters of hierarchical clustering methods. Next, hierarchical clustering methods will merge a pair of clusters if they have a smallest distance or a largest similarity (here, clustering coefficient and neighborhood are used to measure the cluster similarity, and average-linkage method is adopted to merge the clusters). The merging procedure will end when the given number of clusters are obtained. These two semi-supervised hierarchical clustering methods (based on clustering coefficient and neighborhood) are denoted by *ssHC_cc_* and *ssHC_cc_* respectively.

For NG, MCL and MCODE, we adopted a two-stage semi-supervised learning approach with the aid of the prior information (i.e., protein functional pairs). In the first stage, the PPI networks are grouped into a relatively large number of sub-modules by relaxing the parameters of the existing algorithms. For instance, a large value for *s*, the number of splitting steps, will be given for NG algorithm, a large inflation *r* will be set in MCL method and a small cutoff (*t*) will be set in MCODE method. Then, connections between sub-modules are established according to the protein pairs with higher functional relationship in the second stage. Finally, three semi-supervised methods, *ssNG*, *ssMCL* and *ssMCODE*, are designed to mine the functional modules.

## Results and discussion

In this section, we conducted a series of experiments to show how the protein functional pairs improve the performance of the existing modules identification methods (*HC_cc_*, *HC_nb_*, NG, MCL and MCODE) with our proposed prior knowledge based strategy. Yeast PPI networks in MIPs database [33] and GO [30] were used to test the presented methods. In MIPs database, the Yeast PPI networks covers total 12316 protein protein interactions between 4554 proteins, here we ignored the self loop and direction. The identification modules were evaluated by comparing them with the predefined biological complex annotations in MIPs database [33]. Meanwhile, modularity measure [8] was used to select the best parameters.

### Evaluation metrics

#### Modularity

Topology-based modularity metric, proposed by Newman and Girvan [8], can be used to evaluate cluster quality. This metric uses a *k* × *k* symmetric matrix of clusters where each element *d_ij_* represents the fraction of edges that link nodes between clusters *i* and *j* and each *d_ii_* represents the fraction of edges linking vertices within cluster *i.* The modularity measure is given by(7)

Larger value modularity has, better performance the clustering method obtains.

#### Complex annotation measure

Since our goal is to find functional modules from PPI networks, it is necessary to test if the obtained modules correspond to known functional modules. This can be done by validating the modules with the predefined biological annotations from the MIPs database [33]. MIPs provides three domain annotation categories: function annotation, complex annotation and localization annotation. Because function annotation category of MIPs is based on GO and our proposed approach combined the functional information of GO, we used complex category to validate the different identification methods. Merely counting the proteins that share an annotation will be misleading since the underlying distribution of proteins among different annotations is not uniform. Hence, the enrichment analysis are used to calculate the statistical and biological significance of a function module. The enrichment score [51] of a module is the minus log transformation on the geometric mean of *p-*values (i.e., – log(*p* – *value*)) from the enriched annotation terms association with one or more of the module members. The enrichment score essentially shows how the module is involved in the important annotation association with the module members. Probably, the higher the score the more important biology to the gene group.

### Experimental results

#### How much prior information is perfect for learning

**Figure 6:**
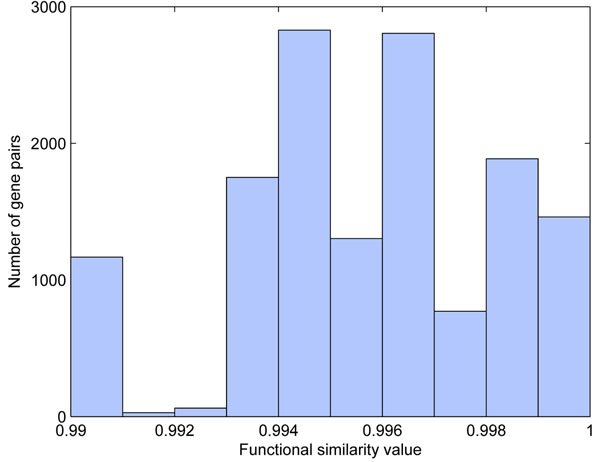
**The Yeast protein functional similarity value ( 0.99) distribution.** The functional similarity value (≥ 0.99) distribution of Yeast protein pairs based on GO (total 14090 unique pairs).

Based on the functional similarity analysis for all Yeast protein pairs in Section , there are total 14090 protein pairs with similarity greater than 0.99, as shown in Figure 6. Among them, 1488 protein pairs (noted as *T*1) have functional similarity value equal to 1, 3375 protein pairs (noted as *T*2) have functional similarity value great than and equal to 0.999, 4146 pairs (noted as *T*3) have similarity value great than and equal to 0.998, 6951 pairs (noted as *T*4) have similarity value great than and equal to 0.997 and etc. In our experiments, we tested the performance of prior knowledge based strategy with the first four sets of protein pairs (named as MYP, MYPT1, MYPT2, MYPT3 and MYPT4 respectively). The first prior knowledge based strategy, combining the protein functional pairs into the PPI networks, was used to show how many protein functional pairs are suitable to be the prior information.

**Figure 7:**
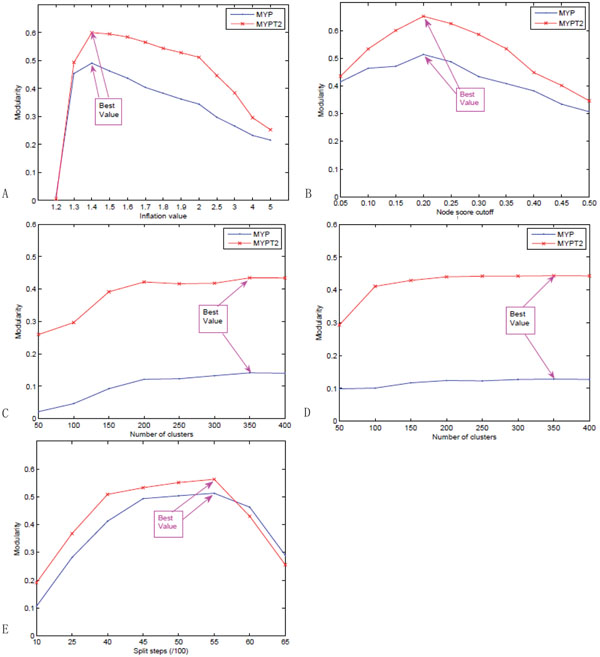
**Impact of the parameter on the modularity of clustering results.** Impact of the parameters on different module identification methods: (a) inflation γ on MCL, (b) node score cutoff *t* on MCODE, (c) number of clusters *k* on *HC_cc_*, (d) number of clusters *k* on *HC_nb_* and (e) splitting steps *t* on *NG.*

**Figure 8:**
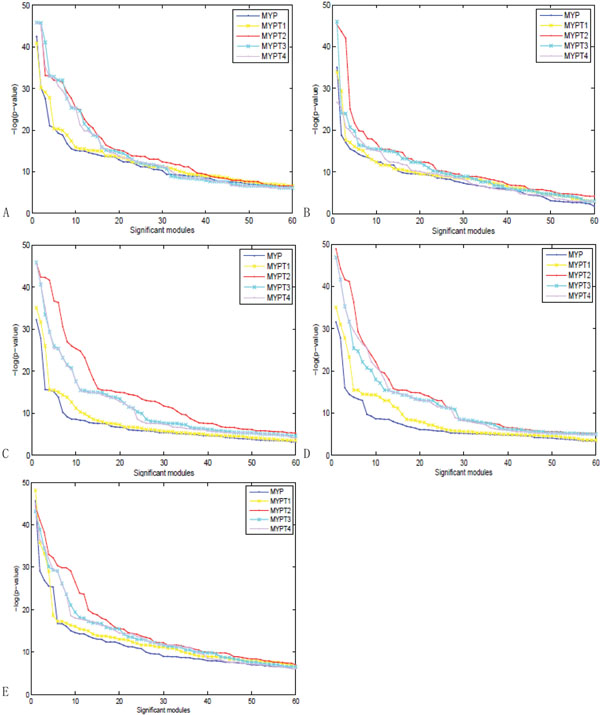
**Comparison of the complex annotation p-value for the functional modules identified by different methods.** The complex annotation *p-*value of the identified functional modules via different methods on MIPs Yeast PPIs with adding different number of functional protein pairs, (a) Hierarchical algorithm based on Clustering coefficient similarity matrix (*HC_cc_*) and (b) Hierarchical algorithm based on Neighborhood similarity (*HC_nb_*), (c) MCL, (d) MCODE and (e) NG.

Five module identification methods were used in our experiments as described in last Section. For each approach, there are some parameters to be predefined, e.g., the inflation factor *r* for MCL, the number of splitting steps *s* in NG method, the node score cutoff *t* in MCODE, and the number of clusters *k* in *HC_cc_*and *HC_nb_.* In this case, the evaluation measure, modularity, was used to validate which parameter value makes the algorithm perform best. For reference, we listed the experimental results for two data sets MYP and MYPT2. The best modularity value for both data sets was obtained at the point 4200, i.e., the number of splitting steps on the modularity of NG method is 4200, as shown in Figure 7(e). Similarly, we can see MCL gets the best performance on modularity when the inflation *r* is equal to 1.4, as shown in Figure 7(a). MCODE got the best performance at the Node score cutoff *t* = 0.2, as shown in Figure 7(b). With the same way, the hierarchical clustering algorithms based on clustering coefficient similarity and neighborhood similarity got their best performance with complete linkage at *k* = 350. That is, the final number of clusters identified by hierarchical algorithms is 350, as shown in Figure 7(c) and Figure 7(d). On the one hand, we experimentally show how the performance of different identification methods are improved by adding different numbers of protein functional pairs to the original protein interaction networks. For each method, five data sets were used, MYP, MYPT1, MYPT2, MYPT3 and MYPT4. They represent the original MIPs Yeast PPIs with 12316 PPIs, adding 1488 protein pairs with similarity = 1, adding 3375 protein pairs with similarity ≥ 0.999, adding 4146 protein pairs with similarity ≥ 0.998, and adding 6951 protein pairs with similarity ≥ 0.997 respectively. Here only the best results will be listed to compare the different algorithms on different data sets. We can consider the –*log*(*p*-value) of the significant modules identified by the corresponding method on one data set. Larger value shows the better performance. Because the smallest number of modules in all experiments is sixty, we showed the top sixty modules for all methods on all data sets. We can find that all algorithms got the best performance on data set MYPT2, adding 3375 protein pairs with similarity ≥ 0.999 on the original protein networks. Even though the other three data sets, MYPT1, MYPT3 and MYPT4, do not make the identification method obtaining the best result, all of them increase the identification performance by comparing with the original PPI networks (MYP). For MYPT3 and MYPT4 adding 4146 protein pairs with similarity (≥ 0.998), and 6951 protein pairs with similarity (≥ 0.997) respectively, the identification results are better than on the original data set MYP, but less than on MYPT2, the reason is that more added protein pairs may add more noise. For MYPT1 adding 1488 protein pairs with similarity (= 1), the identification results are better than on MYP but less than on MYPT2, the reason is that MYPT1 may not have enough functional information. Figure 8 shows the detail results, where each sub-figure represents the experimental results of one identification method, and each line shows the –*log*(*p*-value) of the significant modules identified by the corresponding method on one data set.

#### Comparison of the identification performance

According to the above experimental results, we selected MYP and MYPT2 as the data sets to test the performance of our proposed prior knowledge based strategy. Three parts of experiments were conducted, one for the five existing identification methods (*HC_cc_*, *HC_nb_*, NG, MCL, and MCODE) on the original PPI networks (MYP), the other for these five algorithms on the combined PPI networks (MYPT2), another one for the five proposed semi-supervised *ssHC_cc_*, *ssHC_nb_*, ssNG, ssMCL, and ssMCODE on MYPT2. For the last part of experiments, the protein functional pairs would be taken as the prior information of the identification algorithms.

Table 1 gives a comparison summarization on the first two parts of experimental results. For each algorithm, we listed the number of identified modules (# modules) which are annotated in MIPs complex annotation database, the average – log(*p*-value) (noted as ) and the coverage which is the percentage of proteins which are covered by the annotated modules. From this table, we can see that  on MYPT2 is better than on MYP. Also, we can see that MCODE has the best *p-*value but MCODE only covers a small part of proteins. MCL got the best result both on  and coverage, which is also experimentally proven by Vlasblom and Wodak [44]. Newman-Girvan method got better  than *HC_cc_* and *HC_nb_*, but NG includes less proteins than *HC_cc_* and *HC_nb_.* For two hierarchical clustering methods, it is obvious that clustering coefficient similarity method (*HC_cc_*) is better than neighborhood-based similarity (*HC_nb_*)*.*

**Table 1:** Comparison of different identification methods on the original PPI networks (MYP) and the best extended networks (MYPT2)

	parameter	MYPT2			MYP		

		#modules	AVG -log(*p*-value)	Coverage	#modules	AVG -log(*p*-value)	Coverage
MCL	*r* = 1.4	271	7.8696	0.95249	327	5.44644	0.95623
MCODE	*t* = 0.2	95	11.78031	0.26683	60	8.23775	0.10493
NG	*s* = 4200	279	7.65399	0.79388	292	5.21413	0.81588
*HC_cc_*	*k* = 350	195	7.12497	0.92719	173	4.1128	0.87769
*HC_nb_*	*k* = 350	224	6.65543	0.96706	173	4.0578	0.86120

Table 2 gives the experimental results of our proposed strategy with the aid of prior information. Here, *T*2, the protein pairs with functional similarity (≥ 0.999) were used as the prior information. In ssMCL, ssMCODE and ssNG, *T*2 was used to merge the sub-modules identified by *MCL*, *MCODE* and *NG* respectively, where the initial sub-modules are identified by *MCL* at *r* = 5, by *MCODE* at *t* = 0.05 and by *NG* at *s* = 6500. For hierarchical clustering algorithm *ssHC_cc_* and *ssHC_nb_*, *T*2 was used to construct the initial clusters, and the parameter *k* was set to be 350. Meanwhile, we listed the identification results of the original methods (*MCL*, *MCODE*, *NG*, *HC_cc_* and *HC_nb_*) at the given parameter value to compare with the proposed prior knowledge based methods. Obviously, the proposed strategy obtained better performance in terms of both coverage and complex annotation *p-*value. Even comparing with the best performance of the original methods on MYP in Table 1, our proposed strategy have a comparative performance.

**Table 2:** The performance of prior knowledge based identification methods on PPI networks (MYP) with the aid of protein functional pairs (*T*2)

Parameter	Algo.	MYPT2			Algo.	MYP		
		#modules	–*log*(*p* –* value*)	Coverage		#modules	–*log*(*p* –* value*)	Coverage
*r* = *5*	*ssMCL*	352	7.77136	0.97342	*MCL*	483	4.68541	0.97214
*t* = 0.05	*ssMCODE*	99	11.04522	0.30314	*MCODE*	137	7.66	0.27335
*s* = 6500	*ssNG*	286	6.95497	0.85456	*NG*	522	4.23116	0.83421
*k* = 350	*ssHC_cc_*	201	6.07873	0.92314	*HC_cc_*	173	4.1128	0.87769
*k* = 350	*ssHC_nb_*	220	5.96928	0.93412	*HC_nb_*	173	4.0578	0.86120

**Figure 9:**
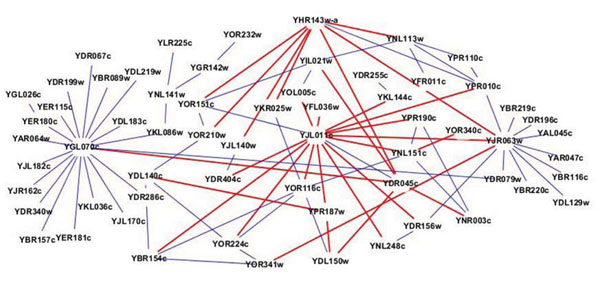
**An example identified by the proposed method.** An example module (with 62 proteins) identified by the proposed method. It is dominated by Complex 550.1.213 and Complex 510.10.

In order to investigate the identified modules, we listed the top ten functional modules identified by the original methods (*MCL*, *MCODE*, *NG*, *HC_cc_* and *HC_nb_*) and our proposed prior knowledge based strategy with the first method (i.e., encoding the prior information (*T*2) into the data representation) in Table 3, 4, 5, 6, 7. For each identified module, we can show the number of proteins it includes (Size), number of total annotated proteins by MIPs complex database (# Annotated), the corresponding complex ID in MIPs database (ComplexID) and the number of genes both in the complex and current module (Hits). We can find that our proposed algorithm can identify functional modules with biological meaning. According to Tables 3-7, we find that there are more common modules detected by *MCL*, *NG* and *HC_cc_* on two data sets MYP and MYPT2 than those detected by *MCODE* and *HC_nb_.* More importantly, we find that there is only one module commonly detected by five different algorithms on MYP, namely, the complexID is 510.190.10. There are around 14 Hits for this module detection. However, there are four modules commonly detected by five different algorithms on MYPT2, namely, the complexIDs are 260.50, 550.1.213, 550.1.147 and 440.30.10. There are totally around 91 Hits for these modules detection. These results show that the enhanced data set can provide a better network for functional module detection. Furthermore, a function module identified by the proposed strategy (ssMCL) is given in Figure 4. The function module with 62 proteins in Figure 9 is dominated by Complex 550.1.213 about ‘probably transcription/DNA maintanance/chromatin structure’ and Complex 510.10 about ‘RNA polymerase’. In this figure, the red line represents the protein pair with higher functional similarity, and the blue line represents the protein pair recorded in MIPs database. ssMCL can successfully find this module because the prior information (protein functional pairs) were added to the original PPI networks. When checking the function modules identified by *MCL* on the original PPI networks (MYP), we did not find this module, while the proteins in the module were divided into different modules. Therefore, we can say prior knowledge based strategy has ability to effectively mine function modules.

**Table 3:** Comparison of complex annotation information for top ten significant modules with best *p*-value identified by *MCL* on MYP and MYPT2.

MYPT2				MYP			
Size	# Annotated	ComplexID	Hits	Size	# Annotated	ComplexID	Hits
28	21	260.50	19	39	34	550.1.149	30
		260.50.10	10			550.2.520	15

156	104	510	61	12	11	60	11
		510.190.10	15			550.1.3	6

62	37	550.1.213	20	40	34	550.1.214	20
		510.10	13			510.190.10	14

55	36	133	19	201	122	140	29
		133.10	10			140.30	20

89	64	550.1.147	31	76	29	260.60	10
		440.30.10	21			550.1.75	10

36	28	290	16	16	12	520.20	8
		290.10	8			520	8

101	55	510.40.20	19	13	9	140.10.20	7
		550.1.209	20			140.10	7

13	12	60	11	148	80	260	24
		550.1.3	6			260.50	11

30	26	550.1.149	21	9	8	550.1.148	8
		550.2.94	13			550.3.56	6

12	9	520.20	9	7	5	290.20.10	5
		550.1.44	5			290.20	5

**Table 4:** Comparison of complex annotation information for top ten significant modules with best *p*-value identified by MCODE on MYP and MYPT2.

MYPT2				MYP			
Size	# Annotated	ComplexID	Hits	Size	# Annotated	ComplexID	Hits
131	105	510	61	14	14	510.190.10	14
		510.1.213	20			510.190.10.20	14

24	24	500.10	21	66	58	60	11
		550.2.380	10			260.60	10

48	47	550.1.147	33	16	9	260.50	8
		440.30.10	24			260	9

160	139	260.50	19	8	8	550.1.148	8
		510.40.20	18			550.3.56	6

49	34	60	11	5	5	510.180.50	5
		550.1.3	6			510.180	5

31	25	550.1.208	13	28	22	140.30	10
		550.2.415	4			550.1.221	6

42	22	520.20	9	5	5	440.12.10	5
		550.1.44	5			550.3.22	5

17	17	550.1.141	10	7	7	510.40	7
		550.3.22	9			510.40.20	6

75	66	220	12	5	5	290.10	5
		550.1.452	9			290	5

16	16	420.50	9	4	4	260.80	4
		420	9			550.1.77	4

**Table 5:** Comparison of complex annotation information for top ten significant modules with best *p*-value identified by *NG* method on MYP and MYPT2.

MYPT2				MYP			
Size	# Annotated	ComplexID	Hits	Size	# Annotated	ComplexID	Hits
43	33	500.10	23	45	39	550.1.149	33
		550.2.380	10			550.2.520	16

43	39	550.1.149	31	14	12	60	11
		510.2.520	15			550.1.3	6

69	36	260.50	19	568	355	140	49
		260	22			140.30	23

216	133	550.1.147	40	67	52	510.190.10	15
		440.30.10	29			510.190.10.20	15

62	46	290	17	14	13	260.60	10
		290.20	9			550.1.75	10

85	49	550.1.213	20	17	10	290.10	7
		510.10	13			290	7

72	37	510.40.20	17	21	15	260.50	9
		550.1.209	19			260	11

14	12	60	11	12	10	510.190.110	7
		550.1.3	6			510.190	7

25	21	410.40	15	12	10	550.1.81	6
		550.1.205	7			160	6

20	16	260.60	10	6	6	510.180.50	5
		550.1.75	10			510.180	5

**Table 6:** Comparison of complex annotation information for top ten significant modules with best *p-value* identified by *HC_cc_* on MYP and MYPT2.

MYPT2				MYP			
Size	# Annotated	ComplexID	Hits	Size	# Annotated	ComplexID	Hits
27	21	260.50	19	17	17	510.190.10	14
		260	20			510.190.10.20	14

42	42	510	39	10	10	260.60	10
		230.20.20	14			550.1.75	10

69	36	510.10	22	12	12	550.1.149	11
		550.2.380	10			550.2.161	5

20	19	510.40.20	18	10	8	510.40	8
		550.1.209	19			510.40.20	7

61	60	550.1.147	33	5	5	510.180.50	5
		440.30.10	24			510.180	5

30	29	550.1.213	20	5	5	440.12.10	5
		510.10	13			550.3.22	5

72	37	220	12	36	27	177	5
		550.1.45	9			550.1.221	5

45	35	2	5	13	11	22	5
		290.20	8			550.1.45	3

14	12	260.60	10	53	31	140	10
		550.1.75	10			140.30.30	6

12	9	520.20	9	3	3	290.20.10	3
		550.1.44	5			290.20	3

**Table 7:** Comparison of complex annotation information for top ten significant modules with best *p-value* identified by *HC_nb_* on MYP and MYPT2.

MYPT2				MYP			
Size	# Annotated	ComplexID	Hits	Size	# Annotated	ComplexID	Hits
26	26	500.10	23	18	18	510.190.10	14
		500	23			510.190.10.20	14

28	23	260.50	19	10	10	260.60	10
		260	20			550.1.75	10

21	19	510.40.20	18	34	23	140.30	11
		550.1.209	19			140.30.30	8

20	19	550.1.147	36	19	19	550.1.138	10
		440.30.10	27			550.2.163	7

30	29	550.1.213	20	5	5	440.12.10	5
		51.10	13			550.3.22	5

53	51	133	19	8	8	470.20	5
		510	30			550.2.153	5

45	35	290	15	6	6	500.10.40	5
		290.20	8			550.1.107	6

13	9	520.20	9	6	5	140.20	5
		520	9			140	5

53	42	140.30	17	73	47	550.1.221	6
		140	17			270.20	7

20	19	550.1.208	13	19	14	510.180.20	4
		550.2.415	4			510.180	5

## Conclusions

In this paper, we presented a prior knowledge based strategy for mining function modules from PPI networks with the aid of GO. The functional protein pairs were extracted according to their functional similarity in GO, and then such pairs were taken as the prior information of the proposed mining methods. Two kinds of prior knowledge based methods were designed: one for encoding the prior information into the data representation, i.e., combining the functional protein pairs and PPI networks to a new PPI networks, and the other for using the prior information as pairwise constraints to supervise the existing ming methods. Experimental results on Yeast PPI networks and GO knowledge resource have shown that our proposed strategy performs well in terms of coverage and complex annotation *p*-value.
